# Micronized Palmitoylethanolamide-Polydatin Reduces the Painful Symptomatology in Patients with Interstitial Cystitis/Bladder Pain Syndrome

**DOI:** 10.1155/2019/9828397

**Published:** 2019-11-11

**Authors:** M. Cervigni, L. Nasta, C. Schievano, N. Lampropoulou, E. Ostardo

**Affiliations:** ^1^Interstitial Cystitis Referral Center, San Carlo di Nancy Hospital, Rome, Italy; ^2^Italian Interstitial Cystitis Association, Rome, Italy; ^3^Innovative Statistical Research, Padua, Italy; ^4^Division of Neurourology and Urodynamic, S. Maria Degli Angeli Hospital, Pordenone, Italy

## Abstract

**Aims:**

To assess the efficacy of a micronized-palmitoylethanolamide-polydatin (m-PEA-Pol) based product on chronic pelvic pain and severity of other symptoms in interstitial cystitis/bladder pain syndrome (IC/BPS) patients refractory to conventional therapies.

**Methods:**

A pilot, open-label bicentric study was carried out involving 32 IC/BPS patients. Chronic, oral m-PEA-Pol treatment lasted 6 months. Bladder pain was evaluated using the visual analog scale, while changes from baseline in other urinary symptoms were evaluated by means of the O'Leary-Sant Interstitial Cystitis Symptom and Problem Index and the Pelvic Pain and Urgency/Frequency (PUF) symptom scale questionnaires. The generalized linear mixed model was used to evaluate significant mean changes across time.

**Results:**

A significant and progressive reduction of pain intensity was observed during m-PEA-Pol treatment (*p* < 0.0001 for reduction over time). The effect was associated with a reduction in severity of patients' symptoms evaluated with the O'Leary-Sant questionnaire (*p*=0.0110 and *p*=0.0014 for cystitis symptoms and problem mean scores, respectively) and the PUF scale (*p*=0.0163 and *p*=0.0005 for symptom and bother mean scores, respectively). m-PEA-Pol therapy elicited a significant reduction over time in the urinary frequency evaluated with voiding diary (*p*=0.0005) and a small but not significant improvement of bladder capacity.

**Conclusions:**

These data highlight the potential benefit of m-PEA-Pol in patients with rare pathology such as IC/BPS and confirm the good safety profile of micronized PEA-based products.

## 1. Introduction

Interstitial cystitis/bladder pain syndrome (IC/BPS) is a chronic disease characterized by persistent pelvic or perineal pain and various voiding symptoms such as nocturia, increased urinary frequency, and urgency. The severity of disease progressively evolves from early to late stage [1]. The prevalence of IC/BPS varies owing to several definitions and methods being used in its estimation. In early studies, IC/BPS was reported to be a rare condition ranging from 10 cases/100,000 in Finland in 1975 to 30/100,000 in the United States in 1987 and 510 cases/100,000 in the United States in 1989 [2]. From these studies, it was already evident that females are more affected. A higher prevalence has been reported in more recent studies, ranging from 52 to 197/100,000 for women and 40 to 70/100,000 for men [3, 4]. At present, IC/BPS is included in the portal for rare diseases and orphan drugs (http://www.orpha.net/consor/cgi-bin/index.php), with an estimated prevalence of 1–5/10,000 in the general population.

The pathogenesis of IC/BPS remains to be fully understood; however, the presence of bladder and lower abdominal pain, bleeding, and thinning of the urothelium is often reported as suggestive of persistent bladder inflammation and urothelial dysfunction [1]. In particular, most studies on humans and animals have focused on role of mast cells as driver of persistent tissue inflammation and pain [5–7]. An increase in mast cell density has been reported in the urothelium and suburothelium areas of patients with IC/BPS [8]. Mast cells not only participate early in disease development in nociceptive pain by sensitization of peripheral nerve fibers but also through persistent stimulation of afferent somatosensory fibers to promote onset of central sensitization and the shift to chronic and neuropathic pain. In this setting, activation of spinal microglia promotes development of spinal neuroinflammation that perpetuates central sensitization [9].

The above evidence suggests that pharmacological control of inflammatory processes may limit bladder damage, as well as peripheral and central sensitization. In this regard, N-acylethanolamines are endogenous lipid mediators able to promote the resolution of inflammation and restore tissue homeostasis. Among N-acylethanolamines, palmitoylethanolamide (PEA), an endogenous fatty acid amide congener of the endocannabinoid anandamide, has been reported to reduce inflammation and pain behaviors in different models of pelvic inflammation [10, 11]. Products based on micronized-(m-) and ultramicronized-(um)-PEA reduce chronic pelvic pain associated to different conditions [12, 13], although no study has been performed to evaluate efficacy of m-PEA-based products on IC/BPS symptoms. This preliminary clinical investigation was carried out to assess the efficacy of a m-PEA-polydatin (m-PEA-Pol) based product on pain, frequency, and urgency in patients suffering from rare pathology such as IC/BPS refractory to conventional therapies.

## 2. Methods

### 2.1. Study Design and Patient Population

An open-label design was adopted in this pilot research. It was performed at San Carlo di Nancy Hospital, Interstitial Cystitis Referral Center, Rome, Italy, and at the S. Maria degli Angeli Hospital, Division of Neurourology and Urodynamics, Pordenone, Italy. Both female and male patients aged 18 years or more with a diagnosis of IC/BPS according to the European Society for the Study of Interstitial Cystitis criteria and unresponsive to first-line noninvasive treatments were enrolled. Eligible patients had to meet at least five of the following criteria: presence of suprapubic, pelvic, urethral, vaginal, or perineal pain for more than 6 months; urinary urgency in more than 50% of micturitions; daytime frequency (>10 times-day) and nighttime (>2 times-night), pain with bladder filling that improves after voiding; presence of glomerulation on cystoscopy with hydrodistention under anesthesia, cystometric bladder capacity ≤300 ml, and sensory urgency (first stimulation ≤100 ml). Only patients able to read, understand, and sign an informed consent were included. Exclusion criteria were as follows: age <18 years, pregnant or breastfeeding women, bladder tumors both benign and malignant, radiation cystitis, vaginitis, symptomatic bladder diverticula, genital herpes in the active phase, bladder and urethral stones, micturition frequency <10 times a day, symptoms present for less than six months, cystometric capacity ≥300 ml with the absence of sensory urgency, the presence of symptoms from less than six months, and intolerance to treatment or active ingredient.

The study was conducted in accordance with the Declaration of Helsinki. All patients signed a written consent for participation in the study. The study was notified to Ethics Committees of both centers. A baseline visit was carried out before treatment start, 15–20 days after the screening visit. The first patient was enrolled on 2010 March, and the last patient completed the study on 2013 July.

### 2.2. m-PEA-Pol Treatment

Eligible patients received a single tablet containing 400 mg m-PEA plus 40 mg polydatin (m-PEA-Pol; Pelvilen Forte, Epitech Group SpA) twice daily for three months followed by once daily for three months.

### 2.3. Clinical Assessments

An initial screening visit was performed to determine patient eligibility and obtain informed consent. In addition to clinical examination and history, a renal and vesical ultrasound scan was carried out on all patients; urodynamic test, cystourethroscopy, and vulvoscopy were performed at the discretion of the clinician. Complete urine analysis and urine culture with ABG as well as vaginal and urethral swabs to detect common germs, protozoa, fungi, mycoplasma, and ureaplasma were carried out as part of the initial screening. Complete urodynamic testing, voiding pressure flow, and cystoscopy were performed before study start.

The primary endpoint was reduction in pelvic pain intensity evaluated by the visual analog scale (VAS) at baseline, during m-PEA-Pol treatment (2, 4, and 6 months from beginning) and two months after stopping treatment (8 months from beginning). The patients were asked to indicate pain intensity on the scale from 0 = ‘‘no pain” to 10 = ‘‘the most painful sensation imaginable.”

Secondary endpoints were changes from baseline in other urinary symptoms recorded using the O'Leary-Sant Interstitial Cystitis Symptom and Problem Index (ICSI/ICPI) [14], the Pelvic Pain and Urgency/Frequency Symptom Scale (PUF) [15], and a 3-day voiding diary. The assessment of safety included the registration of all investigator-assessed adverse events. All study visits were carried out by a clinician.

### 2.4. Statistical Analysis

The results obtained by evaluating VAS pain intensity score, the O'Leary-Sant ICSI, and ICPI and PUF were analyzed using the GLMM (generalized linear mixed model) in order to evaluate mean changes across time. Age, gender, and center were used as covariates. *Post hoc* analysis on GLMM time related curves was performed using the Tukey–Kramer adjusted test for multiple comparisons. The GLMM took into account missing values, correcting the bias due to patients who left the study prematurely. The Wilcoxon signed-rank test was used to compare mean values obtained by VAS pain intensity score, the O'Leary-Sant ICSI, and ICPI and PUF at the end of 6-month m-PEA-Pol treatment vs mean values obtained after two months of discontinuation of treatment. The SAS system (SAS 9.2 System for Windows, SAS Institute, Cary, NC, USA) was used to conduct all statistical analyses.

Data are expressed as mean ± standard error (SE), if not otherwise stated. Results are considered significant for *p* values less than 0.05.

## 3. Results

### 3.1. Patient Characteristics

Thirty-two patients of both sexes with age of 51 ± 12.8 (two patients aged from 21 to 30 years; 5 from 31 to 40; 9 from 41 to 50; 8 from 51 to 60; 6 from 61 to 70; 2 from 71 to 73) between 21 and 73 years were enrolled in the study. Females accounted for about 94% (30/32) of enrolled patients and males the remainder (2/32). Among women, 16 were menopausal, and their age was 60 ± 6.7 years. Patient demographic and baseline clinical characteristics are reported in Table 1.

Overall, 27 patients, including the only two male patients, returned questionnaires properly completed at the end of m-PEA-Pol therapy (84%) and 24 (75%) underwent the last visit two months after stopping treatment.

### 3.2. m-PEA-Pol Effect on Pain Intensity

**Figure 1:**
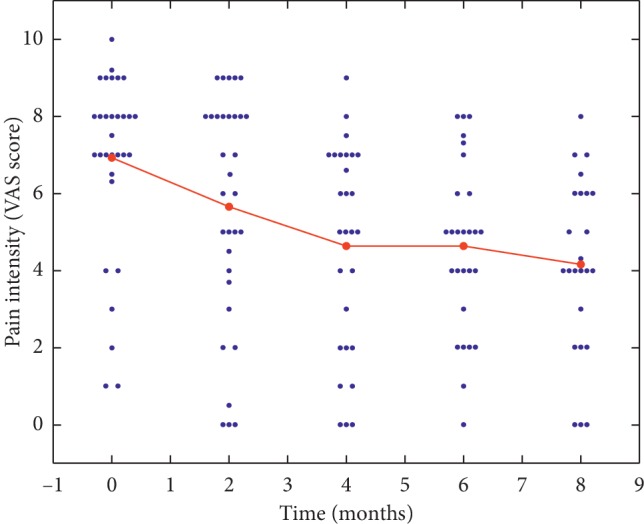
Effect of m-PEA-Pol on pain intensity scores in IC/BPS patients.

During m-PEA-Pol treatment, the average pain intensity score evaluated by VAS decreased from 6.9 ± 0.4 at baseline to 4.6 ± 0.4 at the sixth month in IC/BPS patients. This reduction in mean score was highly significant (*p* < 0.0001) (Figure 1). The variables age, gender, and center did not influence pain intensity average. *Post hoc* analysis of pain intensity score performed on consecutive times showed a significant decrease of estimated mean scores at two months vs baseline (*p*=0.0048), with a further significant decrease at four months vs the previous time (*p*=0.0240). In the interval between four and six months, average scores continued to decrease, albeit more slowly, and comparison of mean scores was not significant (*p*=0.9925). Two months after stopping m-PEA-Pol treatment, the pain intensity score was 4.2 ± 0.5, a value not statistically different from that at the treatment end, as evaluated with the Wilcoxon signed-rank test (*p*=0.2725).

### 3.3. m-PEA-Pol Effect on Interstitial Cystitis Symptoms and Problems

The O'Leary-Sant questionnaire was used to evaluate the severity of patient symptoms and assess progress during m-PEA-Pol treatment. m-PEA-Pol therapy elicited a significant and progressive reduction of both ICSI and ICPI total scores. Average ICSI total score decreased from 11.0 ± 0.8 at baseline to 8.9 ± 0.9 at the sixth month with m-PEA-Pol therapy. Average ICPI total score decreased from 10.3 ± 0.7 at baseline (0) to 7.5 ± 0.9 at the sixth month with m-PEA-Pol therapy (Table 2). Total ICSI and ICPI scores were not influenced by the other variables examined.

*Post hoc* analysis on both ICSI and ICPI total scores performed on consecutive times (2 vs 0; 4 vs 2; and 6 vs 4) did not reveal significant reductions (*p* > 0.05 for all comparisons). Two months after stopping m-PEA-Pol treatment, total ICSI and ICPI scores were 8.9 ± 1.0 and 8.4 ± 0.9, respectively, and not statistically different when compared to those at treatment end (*p* > 0.05 for all comparisons).

Symptoms of pain, urgency, and frequency and the extent to which they bothered the patient were also evaluated by means of the PUF questionnaire. m-PEA-Pol therapy elicited a significant and progressive reduction of both symptoms and bother total scores. Average symptom total score decreased from 12.6 ± 0.8 at baseline (0) to 9.7 ± 1.0 at the sixth month with m-PEA-Pol therapy (Table 3). The reduction was already significant two months after m-PEA-Pol therapy start (*p*=0.013). Average bother total score decreased from 11.0 ± 0.7 at baseline to 7.7 ± 0.9 at sixth month with m-PEA-Pol therapy (Table 3).

Total symptom and bother scores were not influenced by the other variables examined. *Post hoc* analysis on bother total scores performed on consecutive times (2 vs 0; 4 vs 2; and 6 vs 4) did not show significant reductions (*p* > 0.05 for all comparisons). Two months after stopping m-PEA-Pol treatment, symptom and bother total scores were 9.7 ± 1.1 and 8.2 ± 0.9, respectively, and not statistically different from mean values obtained at the end of treatment.

m-PEA-Pol therapy elicited a significant reduction over time in the daily and nocturia urinary frequency evaluated with voiding diary (*p* < 0.0005). A small but insignificant improvement was observed for changes over time of bladder capacity (Table 4).

None of the participants reported adverse events attributable to treatment with m-PEA-Pol.

## 4. Discussion

The main goals of IC/BPS treatment are commonly based on maximizing symptomatic control and quality of life while avoiding adverse events and treatment complications. The present results show that six months of m-PEA-Pol treatment leads to a significant reduction in chronic pelvic pain in patients with IC/BPS. The effect was associated with a reduced severity of patient symptoms evaluated with the O'Leary-Sant and PUF questionnaires, as well as the 3-day voiding diary. Patients did not report any adverse events during and after m-PEA-Pol treatment.

Comparisons between consecutive times of m-PEA-Pol treatment revealed a significant reduction of pain intensity already two months after m-PEA-Pol therapy, with a progressive reduction present at four months. A similar profile was observed for severity of patient symptoms. The progressive reduction of pain scores elicited by m-PEA-Pol confirms preclinical data showing that PEA does not cause tolerance [16]. Moreover, our observations confirm the good safety profile of micronized PEA for long (multimonth) periods, as reported in previous toxicological [17] and clinical studies [18–20]. This aspect provides an important advantage over the prolonged use of common analgesics for chronic pain that can lead to analgesic tolerance, i.e., increasing dosage is needed to maintain efficacy. Such dose adjustments of the latter often increase the risk of serious side effects, leaving the patient unable to continue analgesic therapy.

PEA is an anti-inflammatory and neuroprotective lipid mediator that has been suggested to act on mast cell-glia axis to coordinate inflammatory responses in the peripheral and central nervous systems [9]. Its pharmacological effects are enhanced in the presence of phenols such as polydatin [21], a molecule endowed with antioxidant and anti-inflammatory properties [22]. Although the pathogenetic mechanisms of IC/BPS are not completely understood, long-standing bladder inflammation is considered as a pivotal cause of IC/BPS signs and symptoms [1]. Moreover, as in other chronic conditions associated with chronic pain, dysfunction of immune responses also involve spinal and supraspinal areas [23, 24]. The reduction of chronic pain and severity of other IC/BPS symptoms observed in this study could, conceivably, be related to a local effect on bladder mast cells activity alongside with a control of spinal microglia. The current findings confirm evidence obtained in different experimental studies [11, 25–29].

It is noteworthy that PEA is an anti-inflammatory lipid mediator that acts through different mechanisms, involving distinct membrane (TRPV1, GPR55, etc.) or nuclear (PPARs) receptors as well as nonreceptor mechanisms, suggesting that its pleiotropic action is a critical feature that adapts this molecule for the complexity of chronic pain [10].

The m-PEA-Pol-induced pain intensity reduction confirms the ability of this formulation to exert pain-reducing effects in chronic conditions affecting pelvic organs. This action of m-PEA-Pol has been reported in patients with chronic pain associated to endometriosis [12, 13], perineal pain [30], vulvodynia [31], and irritable bowel syndrome [32]. However, our study provides the first evidence related to chronic bladder pain due to IC/BPS. Importantly, pain intensity reduction was paralleled by a reduction in other common IC/BPS symptoms and bother, as well as urinary frequency, thus suggesting that therapeutic action is probably not exclusively symptomatic. In chronic pain models, PEA not only reduces pain intensity but also behaves as a disease-modifying molecule. In addition, since bladder capacity did not change, it can be postulated that changes in urinary frequency were due to drug effects at spinal and supraspinal level.

The principal limit of the study is the lack of a placebo group so that the possibility that reported outcomes in the patients are due to drug or to the natural course of disease cannot be completely ruled out. However, there is a general consensus on progressive and chronic nature of this complex disease, and a spontaneous healing is unlikely, especially in the participants enrolled in this study who were unresponsive to first-line noninvasive treatments.

These results, although obtained in an open-label pilot survey and on a limited number of patients, inasmuch belonging to rare diseases category, highlight the potential benefit of m-PEA-Pol in patients with IC/BPS and confirm the good safety profile of the micronized PEA-based product. The finding that this group of patients with severe symptoms responded so well to the treatment suggests that this therapy is worthy of additional investigation. In fact, the present data need to be confirmed in a larger multisite placebo-controlled, double-blind clinical study.

## Figures and Tables

**Table 1:** Demographic and baseline clinical characteristics of enrolled patients.

		*n*	%
Age	51 ± 12.8	32	
Sex			
M	2	32	6
F	30	32	94
Menopause	16	29	55
BMI (kg/m^2^)	23.3 ± 5.2	23	
Presence of major comorbidity			
Systemic	16	32	
Gynecologic	2	32	
Previous urogynecological	7	32	
Pain intensity (VAS score)	6.9 ± 2.4		
ICSI score	11.0 ± 4.6		
ICPI score	10.3 ± 3.8		
PUF symptoms	12.6 ± 4.6		
PUF bothers	11.0 ± 3.9		
Bladder capacity (ml)			
Cystomanometric	334.4 ± 116.4	24	
On cystoscopy	655.2 ± 198.1	25	
Voluntary micturition	180.20 ± 19.22	21	
Cystoscopy			
Glomerulations	24	26	92
Bleeding	23	26	88
Hunner lesions	11	25	44

Values are expressed as means ± standard deviation. Data are expressed as means ± SE; *n* = 32 at baseline and two months; 29 and 27 at four and six months, respectively. *p* < 0.0001 for pain intensity score reduction over time.

**Table 2:** Effect of mPEA-Pol on Interstitial Cystitis Symptoms and Problem Index (ICSI and ICPI) evaluated with O'Leary-Sant questionnaire in IC/BPS patients.

	Time (months)				*p*
	0	2	4	6	
ICSI	11.0 ± 0.8	9.9 ± 0.8	8.3 ± 0.9	8.9 ± 0.9	0.0110
ICPI	10.3 ± 0.7	9.1 ± 0.8	8.4 ± 0.9	7.5 ± 0.8	0.0014
Total	21.3 ± 1.4	19.0 ± 1.6	16.8 ± 1.7	16.5 ± 1.7	0.0028

Data are expressed as means ± SE; *n* = 32 at baseline and two months; 29 and 26 at four and six months, respectively. *p* refers to score changes over time.

**Table 3:** Effect of mPEA-POL on the pelvic pain and urgency/frequency symptom evaluated with PUF scale in IC/BPS patients.

	Time (months)				*p*
	0	2	4	6	
Symptom	12.6 ± 0.8	11.0 ± 1.0	10.1 ± 1.0	9.7 ± 1.0	0.0163
Bother	11.0 ± 0.7	9.2 ± 0.8	8.0 ± 0.7	7.7 ± 0.9	0.0005
Total	23.5 ± 1.4	20.2 ± 1.6	18.1 ± 1.6	17.4 ± 1.9	0.0019

Data are expressed as means ± SE; *n* = 32 at baseline and two months; 29 and 26 at four and six months, respectively. *p* refers to score changes over time.

**Table 4:** Effect of mPEA-POL on frequency (daily + nocturia) and bladder capacity evaluated with 3-day voiding diary.

	Time (months)				*p*
	0	2	4	6	
Frequency	12.2 ± 1.2	10.7 ± 1.1	11.6 ± 1.1	10.1 ± 1.1	0.0005
Bladder capacity	180.2 ± 19.2	193.7 ± 20.5	196.8 ± 22.9	208.5 ± 22.5	0.0005

Data are expressed as means ± SE; *n* for frequency = 22, 23, 21, and 19, respectively, at baseline and 2, 4, and 6 months; *n* for bladder capacity = 21, 22, 21, and 19 at baseline and 2, 4, and 6 months, respectively. *p* refers to changes over time.

## Data Availability

The data used to support the findings of this study are included within the article.
